# Clinical benefit of systolic blood pressure within the target range among patients with or without diabetes mellitus: a propensity score-matched analysis of two randomized clinical trials

**DOI:** 10.1186/s12916-022-02407-z

**Published:** 2022-06-20

**Authors:** Chao Li, Kangyu Chen, Guoshuai Shi, Rui Shi, Zhenqiang Wu, Xiaodan Yuan, Vicky Watson, Zhixin Jiang, Hui Mai, Tian Yang, Duolao Wang, Tao Chen

**Affiliations:** 1 Department of Epidemiology and Health Statistics, School of Public Health, Xi’an Jiaotong University Health Science Centre, Xi’an, 710061 China; 2 Department of Cardiology, The First Affiliated Hospital of USTC, Division of Life Sciences and Medicine, University of Science and Technology of China, Hefei, 230001 China; 3 Heart Rhythm Centre, The Royal Brompton and Harefield National Health Service Foundation Trust, National Heart and Lung Institute, Imperial College London, London, SW3 6NP UK; 4 Department of Geriatric Medicine, The University of Auckland, PO Box 93 503, Auckland, New Zealand; 5 Department of Health Education, Affiliated Hospital of Integrated Traditional Chinese and Western Medicine, Nanjing University of Chinese Medicine, Jiangsu Province Academy of Traditional Chinese Medicine, Nanjing, 210028 Jiangsu China; 6 Department of Health Data Sciences, Institute of Population Health, The University of Liverpool, Liverpool, L69 3GB UK; 7 Department of Cardiology, The First Affiliated Hospital of Nanjing Medical University, Jiangsu Province Hospital, Nanjing, 210029 China; 8 Department of Neurology, Central People’s Hospital of Zhanjiang, Zhanjiang, 524000 Guangdong China; 9 Department of Respiratory and Critical Care Medicine, The First Affiliated Hospital of Xi’an Jiaotong University, Xi’an, China; 10 Department of Neurology, Guangdong Key Laboratory of Age-Related Cardiac and Cerebral Diseases, Affiliated Hospital of Guangdong Medical University, Zhanjiang, China; 11 Department of Clinical Sciences, Liverpool School of Tropical Medicine, Pembroke Pl, Liverpool, L3 5QA UK; 12 Department of Public Health, Policy & Systems, Institute of Population Health, Whelan Building, Quadrangle, The University of Liverpool, Liverpool, L69 3GB UK

**Keywords:** Blood pressure, Diabetes mellitus, Cardiovascular disease, Pharmaceutical treatment, Propensity score

## Abstract

**Background:**

Recent guidelines recommended a systolic blood pressure (SBP) target of < 130 mmHg for patients with or without diabetes but without providing a lower bound. Our study aimed to explore whether additional clinical benefits remain at achieved blood pressure (BP) levels below the recommended target.

**Methods:**

We performed a secondary analysis of the Systolic Blood Pressure Intervention Trial (SPRINT) among the non-diabetic population and the Action to Control Cardiovascular Risk in Diabetes BP (ACCORD-BP) trial among diabetic subjects. We used the propensity score method to match patients from the intensive BP group to those from the standard group in each trial. Individuals with different achieved BP levels from the intensive BP group were used as “reference.” For each stratum, the trial-specific primary outcome (i.e., composite outcome of myocardial infarction (MI), acute coronary syndrome not resulting in MI, stroke, acute decompensated heart failure (HF), or cardiovascular death for SPRINT; non-fatal MI, non-fatal stroke, or cardiovascular death for ACCORD-BP) was compared by Cox regression.

**Results:**

A non-linear association was observed between the mean achieved BP and incidence of composite cardiovascular events, regardless of treatment allocation. The significant treatment benefit for primary outcome remained at SBP 110–120 mmHg (hazard ratio, 0.59 [95% CI, 0.46, 0.76] for SPRINT; 0.67 [0.52, 0.88] for ACCORD-BP) and SBP 120–130 mmHg for SPRINT (0.47 [0.34, 0.63]) but not for ACCORD-BP (0.93 [0.70, 1.23]). The results were similar for the secondary outcomes including all-cause mortality, cardiovascular mortality, MI, stroke, and HF. Intensive BP treatment benefits existed among patients maintaining a diastolic BP of 60–70 mmHg but were less distinct.

**Conclusions:**

The treatment benefit persists at as low as SBP 110–120 mmHg irrespective of diabetes status. Achieved very low BP levels appeared to increase cardiovascular events and all-cause mortality.

**Supplementary Information:**

The online version contains supplementary material available at 10.1186/s12916-022-02407-z.

## Background

Numerous studies have shown that lowering blood pressure (BP) in patients with hypertension reduces the risk of BP-related adverse outcomes [1, 2]. However, the optimal BP target for the initiation and treatment with antihypertensive medication remains unresolved and debated [3, 4], particularly in those with diabetes. The contradictory results from the Systolic Blood Pressure Intervention Trial (SPRINT) [5] and the Action to Control Cardiovascular Risk in Diabetes Blood Pressure (ACCORD-BP) [6] leave clinicians with dilemmas and uncertainties, as evident by conflicting guideline recommendations [7, 8]. Although guidelines recommended a tighter BP target for patients with or without diabetes, the lower bound of the BP target was unclear [7, 9]. Additionally, it is unclear whether additional treatment benefits remain at very low achieved BP levels (e.g., 110–120 mmHg).

The results from the analyses of achieved BP in clinical trials and in observational studies have been commonly used to set the BP treatment targets among patients with or without diabetes mellitus [10–12] or coronary heart disease [13, 14]. Such analyses related to clinical outcomes to achieved BP would be attractive as they are based on the patients’ actual BP levels reflecting the clinicians’ real clinical practices rather than the intention-to-treat comparisons of planned randomized groups. However, there are several limitations to these analyses. Unbalanced baseline characteristics (e.g., disease severity or comorbidities) could occur between patients who achieved lower versus higher BP, which may bias the conclusion of a study [15]. Additionally, regression analyses that combine patients from the intervention and control groups may be a surrogate for randomized treatment effects that extend beyond BP, especially when a significant treatment difference exists between the groups in a trial [10, 13].

Partially overlapping achieved BP levels from SPRINT and ACCORD-BP trials provided a unique opportunity to investigate the treatment effects among patients with similar but low achieved BP levels (e.g., Systolic BP [SBP] 110–120 mmHg). Therefore, in the current study, we employed the propensity score method to match patients with different achieved BP levels from the intensive BP group (“reference”) to those from the control group in two randomized clinical trials. On the basis of these comparable data across the groups, our study aimed to explore the optimal achieved BP range to reduce major cardiovascular events in hypertensive patients with or without diabetes.

## Methods

### Trial design and oversight

The current study is a post hoc analysis of limited-access SPRINT [5] and ACCORD-BP [6] BioLINCC datasets obtained from the NIH upon approval. The design and conduct of the randomized, controlled SPRINT and ACCORD-BP trials have been reported previously [5, 6]. Briefly, in the SPRINT trial, 9361 high-risk patients were assigned to either intensive therapy that targeted SBP of lower than 120 mmHg or standard BP treatment that targeted SBP of lower than 140 mmHg, with a median follow-up of 3.26 years [5]. In the ACCORD-BP trial, 4733 high-risk patients were assigned to the intensive or standard control group similar to those used in the SPRINT trial. The mean duration of follow-up was 4.7 years. The trials were approved by the institutional review board or ethics committee at each study site, and all participants provided written informed consent [5, 6]. This analysis was waived for ethical approval by the ethical committee of Liverpool School of Tropical Medicine (No:20-077).

In general, both studies are similar in terms of their study design (e.g., randomized, open-label, multicenter, superiority trials) and treatment algorithms. However, the most notable difference is in the patient populations, with the absence of patients with diabetes in SPRINT and the inclusion of patients with diabetes in ACCORD-BP. Details of the inclusion and exclusion criteria are shown in Additional file 1: Table S1. In our current analysis, we excluded 201 patients from the SPRINT trial and 6 patients from the ACCORD-BP trial because of missing data, lost follow-up, or the primary event occurred in the first 6 months after treatment (Additional file 1: Fig. S1).

### BP measurement and analysis

BP was measured while the participant was seated with the same oscillometric device (model 907, Omron Healthcare, Lake Forest, IL) in both trials. However, an observer remained present during the BP measurements in the ACCORD-BP trial, unlike in the SPRINT trial. BP measurements were taken monthly in the first 3 months and every 3 months thereafter in the SPRINT trial. In the ACCORD-BP trial, patients allocated to the intensive treatment group were seen every month for 4 months and every 2 months thereafter. In the standard treatment group, visits were scheduled at 1 and 4 months and then every 4 months thereafter.

Achieved BP was calculated by averaging the BP values measured from the 6-month visit until the visit before an event or their last visit. We chose the 6-month visit because of the stability of BP values after this visit. Patients were categorized into 4 prespecified strata of achieved BP: < 110 mmHg, 110 to 120 mmHg, 120 to 130 mmHg, and ≥ 130 mmHg for SBP and < 60 mmHg, 60 to 70 mmHg, 70 to 80 mmHg, and ≥ 80 mmHg for diastolic BP (DBP).

### Outcomes

The original primary outcomes for the SPRINT and ACCORD-BP trials were adopted in our analysis. For the SPRINT trial, this was a composite outcome of myocardial infarction (MI), acute coronary syndrome not resulting in MI, stroke, acute decompensated heart failure (HF), or cardiovascular death. For the ACCORD-BP trial, the primary outcome was non-fatal MI, non-fatal stroke, or cardiovascular death. The secondary outcomes in our analysis included cardiovascular death, MI, stroke, HF, and all-cause mortality. A committee blinded to the treatment assignment adjudicated the clinical outcomes in each trial.

### Statistical analysis

We used the propensity score method with replacement to match each patient in the intensive treatment group with a patient in the standard treatment group with similar baseline characteristics. This is to account for potential confounding attributable to the differences in baseline characteristics for each achieved BP stratum in the intensive treatment group with their counterpart in the standard treatment group. A multivariable logistic regression model with the baseline variables shown in Table 1 for the SPRINT trial and Table 2 for the ACCORD-BP trial was used to derive the propensity score for each patient. Greedy matching on propensity scores was performed with a caliper of 0.1. The mean standardized differences of each or overall covariate within each achieved BP stratum are summarized to reflect the balance of covariates before and after propensity score matching.

**Table 1:** Characteristics of patients in the intensive blood pressure control group and propensity score-matched patients from the standard blood pressure control group across the achieved systolic blood pressure strata in the SPRINT trial

	Intention to treat		SBP < 110 mmHg		110 ≤ SBP < 120 mmHg		120 ≤ SBP < 130 mmHg		SBP ≥ 130 mmHg	
	Intensive (*n* = 4579)	Standard (*n* = 4579)	Intensive (*n* = 152)	Standard (*n* = 152)	Intensive (*n* = 2071)	Standard (*n* = 2071)	Intensive (*n* = 1619)	Standard (*n* = 1619)	Intensive (*n* = 636)	Standard (*n* = 636)
**Demographics**										
Age, years	67.88 ± 9.37	67.84 ± 9.43	64.84 ± 9.52	65.53 ± 8.83	66.47 ± 8.89	66.42 ± 9.16	68.70 ± 9.21	68.97 ± 9.32	71.43 ± 9.90	71.95 ± 9.92
Female	1643 (35.88)	1603 (34.99)	58 (38.16)	62 (40.79)	733 (35.39)	762 (36.79)	562 (34.71)	553 (34.16)	261 (41.04)	247 (38.84)
Race										
Black	1346 (29.40)	1389 (30.32)	49 (32.24)	56 (36.84)	570 (27.52)	560 (27.04)	480 (29.65)	461 (28.47)	215 (33.81)	201 (31.60)
White	2645 (57.76)	2649 (57.83)	76 (50.00)	65 (42.76)	1186 (57.32)	1213 (58.57)	968 (59.79)	968 (59.79)	353 (55.50)	370 (58.18)
Hispanic	493 (10.77)	473 (10.32)	25 (16.45)	28 (18.42)	527 (12.72)	256 (12.36)	143 (8.83)	158 (9.76)	48 (7.55)	43 (6.76)
Others	95 (2.07)	70 (1.53)	2 (1.34)	3 (1.97)	85 (2.05)	42 (2.03)	28 (1.73)	32 (1.98)	20 (3.14)	22 (3.46)
**Medical history**										
Clinical CVD	755 (16.49)	758 (16.55)	30 (19.45)	30 (19.45)	316 (15.26)	289 (13.95)	272 (16.80)	293 (18.10)	120 (18.87)	120 (18.87)
CKD	1293 (28.24)	1287 (28.09)	35 (23.03)	32 (21.05)	504 (24.34)	487 (23.52)	474 (29.28)	470 (29.03)	260 (40.88)	243 (38.21)
Dyslipidemia	1941 (42.39)	2034 (44.40)	72 (47.37)	78 (51.32)	884 (42.68)	891 (43.02)	667 (41.20)	669 (41.32)	278 (43.71)	289 (45.44)
Hypertension treatment	4160 (90.85)	4143 (90.44)	140 (92.11)	145 (95.39)	1853 (89.47)	1861 (89.86)	1473 (90.98)	1483 (91.60)	603 (94.81)	600 (94.34)
Aspirin treatment	2361 (51.65)	2304 (50.69)	68 (44.74)	64 (42.11)	1056 (50.99)	1047 (50.56)	883 (54.54)	939 (58.00)	313 (49.21)	310 (48.74)
Current smoking	629 (13.74)	590 (12.90)	35 (23.03)	43 (28.29)	284 (13.71)	270 (13.04)	199 (12.29)	198 (12.23)	87 (13.68)	95 (14.94)
Current drinking	1632 (35.77)	1607 (35.20)	55 (36.18)	46 (30.29)	733 (35.39)	753 (36.36)	561 (34.65)	537 (33.17)	253 (39.78)	257 (40.41)
**Biometric and laboratory data**										
10-year risk for CVD, %	24.78 ± 12.59	24.78 ± 12.44	21.25 ± 11.45	22.42 ± 11.81	22.91 ± 11.76	22.74 ± 11.66	25.73 ± 12.42	25.70 ± 12.17	28.38 ± 13.96	29.96 ± 13.20
BMI, kg/m^2^	29.87 ± 5.68	29.74 ± 5.59	31.90 ± 6.31	32.03 ± 7.23	30.38 ± 5.64	30.40 ± 5.57	29.50 ± 5.53	29.60 ± 5.50	28.52 ± 5.49	28.52 ± 5.53
SBP, mmHg	139.60 ± 15.74	139.63 ± 15.38	132.63 ± 17.71	134.01 ± 13.31	136.85 ± 14.48	136.88 ± 14.66	141.28 ± 15.67	140.25 ± 15.59	146.33 ± 16.44	146.84 ± 16.60
DBP, mmHg	78.24 ± 11.89	78.07 ± 11.93	78.78 ± 12.81	78.69 ± 11.14	78.80 ± 11.05	78.84 ± 11.75	77.90 ± 12.26	77.40 ± 11.77	76.94 ± 13.09	76.62 ± 12.24
BLU, mmol/L	6.67 ± 2.40	6.72 ± 2.39	6.48 ± 2.22	6.43 ± 1.91	6.48 ± 2.22	6.49 ± 2.11	6.69 ± 2.35	6.70 ± 2.41	7.29 ± 3.00	7.13 ± 2.91
Chloride, mmol/L	102.92 ± 2.90	102.93 ± 2.84	103.19 ± 3.21	103.11 ± 2.66	103.12 ± 2.73	103.07 ± 2.85	102.72 ± 2.93	102.75 ± 2.96	102.80 ± 3.20	102.99 ± 2.84
Creatinine, μmmol/L	94.74 ± 30.38	95.17 ± 29.69	90.25 ± 27.45	89.58 ± 27.71	91.67 ± 26.91	91.05 ± 25.81	95.90 ± 30.94	95.30 ± 30.74	102.58 ± 36.99	100.51 ± 34.76
Heart rate	66.18 ± 11.49	66.26 ± 11.59	66.60 ± 11.75	67.62 ± 11.34	66.71 ± 11.36	66.97 ± 11.47	65.81 ± 11.53	66.15 ± 11.25	64.66 ± 11.10	67.49 ± 21.99
eGFR, mL/min/1.73 m^2^	71.90 ± 20.68	71.69 ± 20.44	76.88 ± 23.75	76.94 ± 22.42	74.01 ± 20.14	74.00 ± 19.92	70.83 ± 19.99	71.21 ± 20.23	66.15 ± 21.46	67.49 ± 21.99
Glucose, mmol/L	5.54 ± 0.77	5.53 ± 0.75	5.49 ± 0.68	5.42 ± 0.70	5.56 ± 0.77	5.61 ± 0.87	5.53 ± 0.77	5.55 ± 0.80	5.50 ± 0.78	5.51 ± 0.76
HDL-C, mmol/L	1.37 ± 0.37	1.37 ± 0.38	1.33 ± 0.35	1.34 ± 0.33	1.34 ± 0.34	1.34 ± 0.35	1.39 ± 0.39	1.38 ± 0.39	1.44 ± 0.40	1.41 ± 0.35
LDL-C, mmol/L	2.91 ± 0.92	2.90 ± 0.90	2.93 ± 0.89	2.89 ± 0.92	2.91 ± 0.91	2.93 ± 0.93	2.92 ± 0.93	2.91 ± 0.90	2.87 ± 0.92	2.92 ± 0.93
Potassium, mmol/L	4.21 ± 0.44	4.20 ± 0.45	4.19 ± 0.54	4.20 ± 0.72	4.19 ± 0.42	4.18 ± 0.43	4.21 ± 0.42	4.21 ± 0.48	4.23 ± 0.48	4.22 ± 0.45
Sodium, mmol/L	140.12 ± 2.46	140.15 ± 2.42	140.14 ± 2.53	140.32 ± 2.20	140.31 ± 2.25	140.26 ± 2.43	139.99 ± 2.57	139.95 ± 2.53	139.95 ± 2.75	140.17 ± 2.43
TC, mmol/L	4.92 ± 1.07	4.91 ± 1.06	4.88 ± 1.05	4.81 ± 1.07	4.89 ± 1.05	4.91 ± 1.06	4.94 ± 1.07	4.91 ± 1.05	4.89 ± 1.05	4.93 ± 1.10
Triglycerides, mmol/L	1.37 ± 0.95	1.40 ± 1.04	1.29 ± 0.55	1.23 ± 0.61	1.36 ± 0.68	1.37 ± 0.71	1.32 ± 0.68	1.33 ± 0.69	1.23 ± 0.61	1.27 ± 0.66
**Post-baseline characteristics**										
Achieved SBP, mmHg	121.72 ± 8.78	135.51 ± 7.45	107.05 ± 2.65	134.20 ± 8.42	116.09 ± 2.54	134.95 ± 7.41	123.89 ± 2.72	136.07 ± 7.19	137.42 ± 8.37	138.09 ± 8.20
Achieved DBP, mmHg	67.98 ± 8.43	75.11 ± 9.17	64.42 ± 7.70	77.65 ± 9.25	67.01 ± 7.08	76.25 ± 8.89	68.14 ± 8.42	74.65 ± 8.97	71.84 ± 9.86	71.48 ± 11.18

Categorical variables are reported as the percentage with the characteristic. Continuous variables are reported as mean ± SD

Baseline covariables listed in the table are included in the logistic model to calculate the propensity score of each patient from the SPRINT trial

*BLU* blood urea nitrogen, *BMI* body mass index, *CKD* chronic kidney disease, *CVD* cardiovascular disease, *DBP* diastolic blood pressure, *eGFR* estimated glomerular filtration rate, *HLD-C* high-density lipoprotein cholesterol, *LDL-C* low-density lipoprotein cholesterol, *SBP* systolic blood pressure, *TC* total cholesterol

**Table 2:** Characteristics of patients in the intensive blood pressure control group and propensity score-matched patients from the standard blood pressure control group across the achieved systolic blood pressure strata in the ACCORD trial

	Intention to treat		SBP < 110 mmHg		110 ≤ SBP < 120 mmHg		120 ≤ SBP < 130 mmHg		SBP ≥ 130 mmHg	
	Intensive (*n* = 2359)	Standard (*n* = 2368)	Intensive (*n* = 173)	Standard (*n* = 173)	Intensive (*n* = 1169)	Standard (*n* = 1169)	Intensive (*n* = 676)	Standard (*n* = 676)	Intensive (*n* = 310)	Standard (*n* = 310)
**Demographics**										
Age, years	62.72 ± 6.60	62.75 ± 6.76	62.15 ± 6.81	61.28 ± 6.06	62.03 ± 6.50	62.25 ± 6.63	63.03 ± 6.56	63.56 ± 6.90	64.98 ± 6.47	65.01 ± 6.55
Female	1127 (47.77)	1129 (47.68)	104 (60.12)	107 (61.85)	552 (47.22)	519 (44.40)	316 (46.75)	291 (43.05)	171 (55.16)	169 (54.52)
Race										
Black	546 (23.15)	577 (24.37)	28 (16.18)	40 (723.12)	214 (18.31)	224 (19.16)	176 (26.04)	182 (26.92)	121 (39.03)	124 (40.00)
White	1411 (59.81)	1368 (57.77)	127 (73.41)	125 (70.62)	741 (63.39)	731 (62.53)	374 (55.33)	386 (57.10)	136 (43.87)	132 (42.58)
Hispanic	160 (6.78)	170 (7.18)	5 (2.89)	3 (1.73)	78 (6.67)	75 (6.42)	51 (7.54)	48 (7.10)	28 (9.03)	32 (10.32)
Others	242 (10.26)	253 (10.68)	13 (7.51)	9 (5.20)	136 (11.63)	139 (11.89)	66 (9.76)	69 (10.21)	22 (7.10)	25 (8.06)
Randomized glycemic treatment	1177 (49.89)	1193 (50.38)	93 (53.76)	85 (49.13)	573 (49.02)	563 (48.16)	341 (50.44)	335 (49.56)	163 (52.58)	162 (52.26)
**Medical history**										
Clinical CVD	803 (34.04)	788 (33.28)	65 (37.57)	68 (39.31)	379 (32.42)	372 (31.82)	224 (33.14)	235 (34.76)	118 (38.06)	120 (38.71)
Clinical heart disease	658 (27.89)	657 (27.74)	61 (35.26)	57 (32.95)	327 (28.31)	331 (28.31)	181 (26.78)	199 (29.44)	78 (25.16)	85 (27.42)
Dyslipidemia	1629 (69.06)	1670 (70.52)	129 (74.57)	122 (70.52)	833 (71.26)	835 (71.43)	443 (65.53)	427 (63.17)	203 (65.48)	206 (66.45)
Hypertension treatment	2082 (88.26)	2068 (87.33)	153 (88.44)	152 (87.86)	1011 (86.48)	996 (85.20)	607 (89.79)	616 (91.12)	281 (90.65)	281 (90.65)
Dyslipidemia treatment	1906 (80.80)	1892 (79.90)	144 (83.24)	149 (86.13)	977 (82.87)	969 (82.89)	537 (79.44)	532 (78.70)	233 (75.16)	241 (77.74)
Current smoking	971 (41.16)	977 (41.26)	76 (43.93)	71 (41.04)	502 (42.94)	500 (42.77)	276 (40.83)	294 (43.49)	104 (33.55)	96 (30.97)
Current drinking	561 (23.78)	544 (22.97)	33 (19.08)	39 (22.54)	285 (24.38)	283 (24.21)	169 (25.00)	196 (28.99)	67 (21.61)	73 (23.55)
**Biometric and laboratory data**										
BMI, kg/m^2^	32.19 ± 5.60	32.10 ± 5.38	32.62 ± 5.40	32.50 ± 5.37	32.07 ± 5.54	32.31 ± 5.49	32.29 ± 5.66	31.59 ± 5.16	31.97 ± 5.78	32.26 ± 5.56
SBP, mmHg	139.02 ± 16.12	139.34 ± 15.54	129.53 ± 15.18	129.79 ± 13.50	136.08 ± 14.28	135.52 ± 14.37	141.69 ± 16.53	141.53 ± 16.36	149.74 ± 15.77	149.79 ± 17.05
DBP, mmHg	75.93 ± 10.56	75.98 ± 10.23	73.88 ± 10.14	75.00 ± 9.48	75.65 ± 9.74	75.07 ± 9.85	76.43 ± 11.56	76.65 ± 10.67	77.16 ± 11.55	77.21 ± 10.31
Waist, cm	106.02 ± 14.08	105.36 ± 13.26	106.71 ± 14.24	106.09 ± 12.62	105.97 ± 13.96	106.28 ± 12.94	106.45 ± 13.96	105.39 ± 13.15	104.79 ± 14.42	105.33 ± 13.73
Creatinine, μmmol/L	79.22 ± 21.05	79.22 ± 20.85	74.76 ± 19.34	74.71 ± 18.42	77.43 ± 20.50	76.13 ± 19.05	80.93 ± 20.49	82.29 ± 21.15	85.23 ± 23.43	87.03 ± 22.48
CPK, mg/dL	137.45 ± 119.35	146.51 ± 145.12	120.78 ± 111.22	117.60 ± 74.95	132.10 ± 101.81	132.92 ± 109.03	149.17 ± 138.98	142.97 ± 114.91	143.60 ± 139.06	155.22 ± 132.46
eGFR, mL/min/1.73 m^2^	91.57 ± 30.30	91.63 ± 27.13	91.96 ± 26.83	92.17 ± 22.47	93.84 ± 25.89	95.16 ± 29.29	89.88 ± 38.07	89.41 ± 28.74	85.34 ± 26.10	84.49 ± 28.84
Glucose, mmol/L	9.86 ± 3.23	9.70 ± 3.23	9.35 ± 2.79	9.23 ± 2.35	9.75 ± 3.10	9.64 ± 3.10	10.08 ± 3.32	10.04 ± 3.48	10.00 ± 3.71	10.11 ± 3.44
HDL-C, mmol/L	1.19 ± 0.34	1.20 ± 0.36	1.20 ± 0.35	1.20 ± 0.34	1.19 ± 0.33	1.19 ± 0.36	1.19 ± 0.36	1.22 ± 0.39	1.20 ± 0.33	1.22 ± 0.34
LDL-C, mmol/L	2.87 ± 0.97	2.81 ± 0.93	2.93 ± 1.04	3.00 ± 0.95	2.83 ± 0.96	2.78 ± 0.99	2.87 ± 0.94	2.84 ± 0.96	3.03 ± 0.99	2.88 ± 0.92
Potassium, mmol/L	4.47 ± 0.47	4.47 ± 0.57	4.48 ± 0.40	4.44 ± 0.43	4.46 ± 0.47	4.46 ± 0.44	4.49 ± 0.45	4.52 ± 0.49	4.48 ± 0.50	4.43 ± 0.47
TC, mmol/L	5.02 ± 1.17	4.95 ± 1.15	5.06 ± 1.17	5.08 ± 1.09	4.97 ± 1.17	4.94 ± 1.25	5.03 ± 1.12	4.99 ± 1.14	5.13 ± 1.17	4.93 ± 1.04
Triglycerides, mmol/L	2.14 ± 1.96	2.10 ± 2.03	2.03 ± 1.45	1.96 ± 1.27	2.14 ± 1.93	2.21 ± 2.66	2.21 ± 1.96	2.13 ± 2.13	2.02 ± 1.75	1.84 ± 1.40
**Post-baseline characteristics**										
Achieved SBP, mmHg	120.43 ± 9.66	133.83 ± 9.40	107.04 ± 3.10	131.70 ± 9.20	115.49 ± 2.57	132.54 ± 8.66	124.04 ± 2.82	134.02 ± 10.13	138.95 ± 9.55	136.36 ± 10.20
Achieved DBP, mmHg	65.39 ± 7.48	71.31 ± 8.01	61.85 ± 5.74	72.07 ± 7.53	64.74 ± 6.69	71.28 ± 7.71	65.82 ± 7.53	70.49 ± 8.78	68.91 ± 9.49	70.14 ± 8.80

Categorical variables are reported as percentage with the characteristic. Continuous variables are reported as mean ± SD

Baseline covariables listed in the table are included in the logistic model to calculate the propensity score of each patient from the ACCORD trial

*BMI* body mass index, *CPK* creatine phosphokinase, *CVD* cardiovascular disease, *DBP* diastolic blood pressure, *eGFR* estimated glomerular filtration rate, *HLD-C* high-density lipoprotein cholesterol, *LDL-C* low-density lipoprotein cholesterol, *SBP* systolic blood pressure, *TC* total cholesterol

We used the Cox model to calculate the treatment effect according to each achieved BP stratum, with a robust variance estimator to account for the clustering within matched sets. The incidence rate of each clinical outcome for each trial (events per 100 patient-years) after 6 months is summarized. To further explore the relationship between achieved BP and primary outcome in both trials, we employed spline analysis within the Cox regression model among the intensive BP control group, which included SBP and DBP as natural cubic splines to account for a continuous non-linear functional dependence. We specified 140 mmHg and 90 mmHg as the reference value for SBP and DBP, respectively. Spline knots were placed at the 10th, 30th, 70th, and 90th centiles of the overall distribution of SBP and DBP.

We also performed a series of sensitivity analyses including repeated analysis for the secondary outcome and the unmatched analysis (compared with all patients in the standard treatment group) to calculate hazard ratios (HRs) and 95% confidence intervals (CIs) of treatment effect within each achieved BP stratum. All analyses were performed using STATA version 15.0 (Stata Corporation).

## Results

Tables 1 and 2 and Additional file 1: Tables S2 and S3 show the baseline characteristics for the patients from each of the 4 strata of achieved SBP or DBP levels in the intensive treatment group and their matching patients from the standard treatment group. In general, baseline characteristics were similar between intensive and standard BP control groups across each of the 4 strata of achieved SBP or DBP levels. This was supported by balance diagnostics tests for overall and individual covariables, where the standardized differences were generally within 10% across each stratum of achieved SBP or DBP levels after matching, particularly for those from the middle BP range (Additional file 1: Table S4, Figs. S2 and S3).

**Fig. 1:**
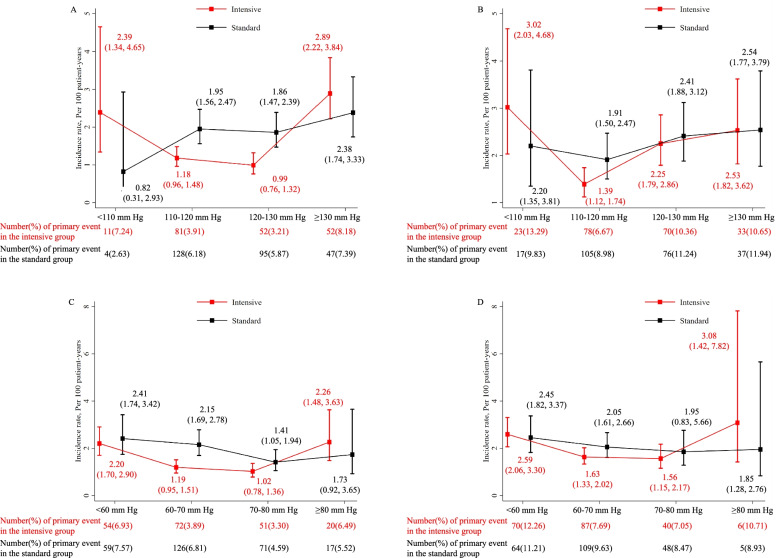
Incidence rates and 95% confidence interval in the intensive blood pressure control group across achieved SBP and DBP strata for the primary outcome of SPRINT and ACCORD trials. Incidence rate per 100 patient-years, compared with matched patients in the standard control group, is shown in the following order: **A** achieved SBP in the SPRINT trial, **B** achieved SBP in the ACCORD trial, **C** achieved DBP in the SPRINT trial, and **D** achieved DBP in the ACCORD trial

**Fig. 2:**
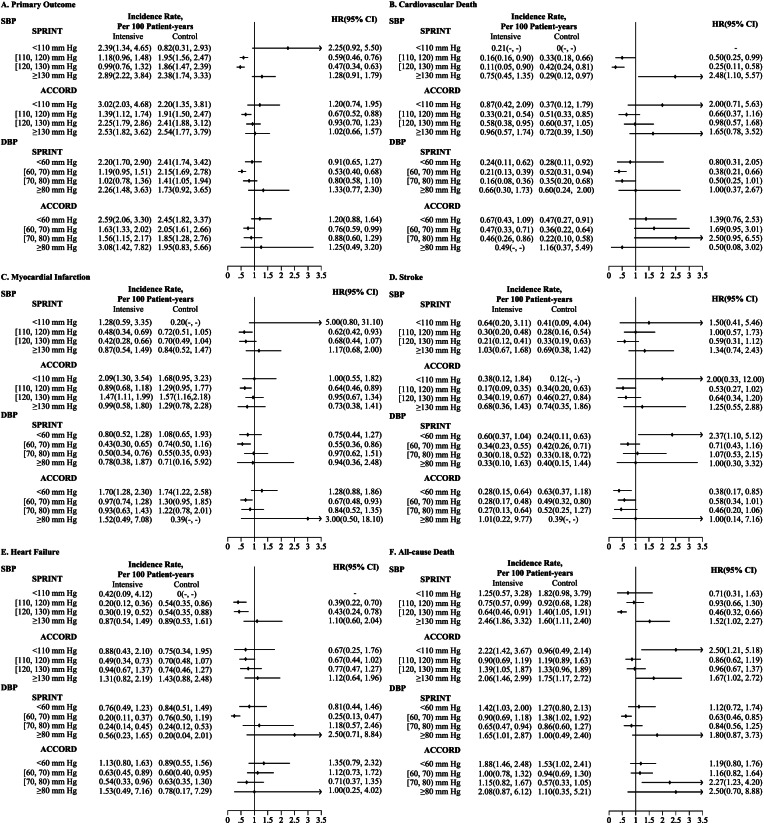
Incidence rate and HRs in the intensive and standard blood pressure control groups across achieved SBP and DBP strata for  primary outcome and secondary outcomes. Incidence rate per 100 patient-years and HRs of intensive blood pressure treatment effect, compared with matched patients in the standard blood pressure control group, in SPRINT and ACCORD studies according to the levels of achieved SBP and DBP with **A** primary outcome, **B** cardiovascular death, **C** myocardial infarction, **D** stroke, **E** heart failure, and **F** all-cause death. The propensity score was calculated by fitting the logistic regression model by adding the variables of age, sex, race, history of clinical CVD, history of CKD, history of dyslipidemia, history of hypertensive treatment, history of aspirin treatment, current smoking, current drinking, 10-year risk for CVD, BMI, SBP, DBP, BLU, chloride, creatinine, heart rate, eGFR, glucose, HDL-C, LDL-C, potassium, sodium, total cholesterol, and triglycerides in the SPRINT study; age, sex, race, history of clinical CVD, history of heart disease, history of dyslipidemia, history of hypertensive treatment, history of dyslipidemia treatment, current smoking, current drinking, BMI, SBP, DBP, waist, creatinine, CPK, eGFR, glucose, HDL-C, LDL-C, potassium, total cholesterol, and triglycerides in the ACCORD trial

A non-linear relationship between achieved BP and incidence rate of primary outcome was found in both trials with a higher incidence rate at higher and lower BP values, particularly among the intensive treatment group (Fig. 1). For the SBP, we observed a plateau range for the event rate (per 100 patient-years) in the SPRINT trial at 110–120 mmHg (1.18; 95% CI 0.96,1.48) and 120–130 mmHg (0.99; 0.76, 1.32) among the intensive BP group, which was lower than their corresponding rates [1.95 (1.56, 2.47) and 1.86 (1.47, 2.39)] among the standard treatment group (Fig. 1A). Our results from the Cox model further demonstrated that intensive BP lowering significantly reduced the incidence of the primary outcome in the strata of 110–120 mmHg (HR = 0.59; 95% CI 0.46, 0.76) and 120–130 mmHg (0.4; 0.34, 0.63) (Fig. 2A) in the SPRINT trial. Likewise, we found the lowest event rate was at 110–120 mmHg in the ACCORD-BP trial, and participants from the intensive group have a lower event rate than those from the standard treatment group [(1.39; 1.12, 1.74) versus (1.91; 1.50, 2.47), HR = 0.67; 0.52, 0.88] but did not achieve statistical significant at 120–130 mmHg [(2.25; 1.79, 2.86) versus (2.41; 1.88, 3.12), HR = 0.93; 0.70, 1.23] (Figs. 1B and 2A). The same pattern was found for DBP with the low incidence rates at 60–80 mmHg in both trials (Fig. 1C, D). However, the treatment effect is only significant in the strata of 60–70 mmHg (0.53; 0.40, 0.68 for SPRINT; 0.76; 0.59, 0.99 for ACCORD-BP) (Fig. 2A). Additionally, our findings from both trials indicated that the benefit of the intensive BP lowering may not retain at very low achieved SBP (< 110 mmHg) with a relatively higher incidence rate in the intensive treatment group.

**Fig. 3:**
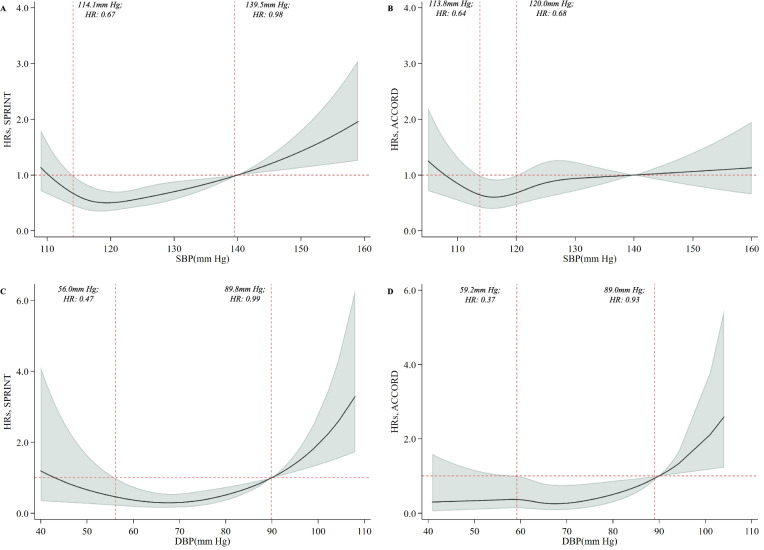
Spline analyses of achieved SBP and DBP in the intensive blood pressure treatment group. HRs for the combined primary outcome (shadow represents the upper and lower bounds of 95% CI) are relative to 140 mmHg for SBP and 90 mmHg for DBP. Knots are placed at the 10th, 30th, 70th, and 90th centiles of achieved SBP and DBP. The multivariable model was adjusted for the variables of age, sex, race, history of clinical CVD, history of hypertensive treatment, history of dyslipidemia treatment, current smoking, current drinking, BMI, SBP, DBP, eGFR, glucose, HDL-C, LDL-C, potassium, total cholesterol, and triglycerides

The results from individual cardiovascular events and mortality as well as the unmatching analysis (comparing with all patients in the standard treatment group) generally confirmed the findings for the primary outcome, especially for the achieved BP at 110–130 mmHg for SBP and 60–80 mmHg for DBP (Fig. 2B–E, Additional file 1: Fig. S4). To validate our results, we further employed a spline analysis among the intensive treatment group and found a non-linear relationship between achieved SBP and the primary outcome (Fig. 3). The model indicated that achieved SBP from 114.1 to 139.5 mmHg was significantly associated with the decreased risk of having a primary outcome in the SPRINT trial and 113.8–120.0 mmHg in the ACCORD-BP trial. Meanwhile, the J-shaped curve was noted for achieved DBP with a decreased risk at 56.0–89.8 mmHg for the SPRINT trial and 59.2–89.0 mmHg for the ACCORD-BP trial. Additional file 1: Table S5 summarizes the incidence of safety outcomes (only available in the SPRINT trial) including any serious adverse events and serious adverse events associated with hypotension, syncope, electrolyte abnormality, acute kidney injury, or acute kidney failure across the strata of achieved SBP and DBP levels. Those with the lowest achieved DBP level (DBP < 60 mmHg) and highest SBP level (SBP ≥ 130 mmHg) had the highest incidence of serious adverse events, but no evidence demonstrated heterogeneity of the effects of the treatment by achieved SBP and DBP levels.

## Discussion

On the basis of this secondary analysis of the SPRINT and ACCORD trials, our study demonstrated that the treatment benefit in reducing major cardiovascular events persists at as low as achieved SBP between 110 and 120 mmHg irrespective of diabetes status. In contrast, a reduction of SBP to less than 110 mmHg or higher than 130 appeared to increase the risk of composite endpoints of cardiovascular disease (CVD) events, which was supported by a spline analysis in the intensive treatment group. A similar pattern was observed for achieved DBP at 60–70 mmHg in both SPRINT and ACCORD trials, although achieved DBP values had diverse benefits for different outcomes. There was no apparent excess risk of adverse events in patients who achieved SBP at 110–130 mmHg and DBP at 60–80 mmHg from available SPRINT trial safety data.

Analysis based on the achieved BP, in comparison with the randomized BP target as per the intention-to-treat principle, is attractive and has commonly been used to set BP treatment targets [13, 16, 17]. Unlike previous analyses which relate achieved BP levels to the incidence of cardiovascular outcomes, but are subject to limitations of confounding [15, 18, 19], our study adopted the propensity score matching to compare patients in 4 strata of achieved SBP or DBP with intensive BP treatment to patients in the standard BP treatment with similar baseline characteristics. After matching, the results from the balance diagnostics clearly showed the differences in baseline covariables between the intervention and control groups were mitigated. Our analysis indicated a non-linear association between achieved BP and incidence of the combined CVD events among both intensive and standard BP groups. This was further supported by our spline analysis, where a range of SBP 113.8–120.0 mmHg and 114.1–139.5 mmHg was significantly associated with a lower risk of cardiovascular outcomes for patients with diabetes in the ACCORD trial and without diabetes in the SPRINT trial, respectively. The results from our study are consistent with several prior reports [10, 13, 17, 18, 20], showing that high or low treatment BP levels are associated with an increased risk of cardiovascular outcomes and death. This J- or U-shaped relationship has been a matter of concern endorsed by many experts, and the optimal BP target for hypertensive patients with or without diabetes remains inconclusive [21–24].

Although researchers argued that inadequate perfusion of organs from very low BP would result in a higher rate of adverse outcomes, treatment might offer additional protection at some level of low BP. In our current analysis, we demonstrated that the benefit of the intensive SBP lowering could exist at achieved SBP (110–130 mmHg) or DBP (60–80 mmHg) but might be associated with a higher risk of CVD outcome or mortality for very low achieved BPs (< 110 mmHg or < 60 mmHg). These findings persisted in our sensitivity analyses without matching and for our secondary outcomes (e.g., stroke, MI, HF, and mortality). Our results were generally in line with a recent large-scale analysis of 48 randomized trials showing that drugs to lower BP are similarly effective for primary and secondary prevention of major cardiovascular disease over the whole BP range, even if BP is in an apparently healthy range (< 120 mmHg) [25], regardless of age [26]. This was also supported by some early studies [1, 18, 27]. However, these studies did not evaluate the treatment benefit at a further lower BP (e.g., 110–120 mmHg). It is noted that in those with diabetes, intensive SBP lowering could significantly decrease the risk of cardiovascular composite end point at achieved SBP (110–120 mmHg), which needs further evaluations.

Although our study confirmed the prior findings of the non-linear association (e.g., J or U curve) between achieved BP and incidence of the combined CVD events, there are several strengths in our study including the assessment of the treatment benefit from achieved BPs (e.g., 110–120 mmHg, 120–130 mmHg) with their matched comparators rather than solely relied on the BP-CVD risk association. This study also has some limitations. First, the sample size and the number of events in the subgroups with the lowest achieved SBP (< 110 mmHg) were small, which prevent us to reveal a conclusive association between the groups among this stratum. Second, this is a post hoc analysis of two clinical trials data; thus, the results from our analyses based on post-randomized data (such as achieved level of BP) may still be subject to residual confounding and reverse causality. Third, selection bias may occur due to matching but our unmatching analysis provided consistent results with those from propensity score matching analysis. Finally, different methods were used to measure BP in SPRINT and ACCORD-BP. Studies have shown automated BP measurements performed in the absence of an observer could yield significantly lower SBP and DBP values compared with conventional BP measurements [28, 29]. However, we find a similar pattern of the association between achieved BP and cardiovascular events or mortality in both trials, even with a different composite primary outcome.

## Conclusions

Our analysis revealed that the treatment benefit persists at as low as SBP 110–120 mmHg irrespective of diabetes status. Achieved very low BP levels appeared to increase cardiovascular events and all-cause mortality.

## Supplementary Information

**Additional file 1: Table S1.** Inclusion and exclusion criteria of SPRINT and ACCORD trial. **Table S2.** Characteristics of patients in the intensive blood pressure control group and propensity score-matched patients from the standard blood pressure control group across the achieved diastolic blood pressure strata in the SPRINT trial. **Table S3.** Baseline characteristics of patients in the intensive blood pressure control group and propensity score-matched patients from the standard blood pressure control group across the achieved diastolic blood pressure strata in the ACCORD trial. **Table S4.** Mean standardized difference for all of the covariates used in the propensity score-matched method. **Table S5.** Incidence (per 100 person-years) of serious adverse events in the intensive and standard blood pressure control groups across achieved SBP and DBP strata (SPRINT trial). **Fig. S1.** Data exclusion in the present analysis. **Fig. S2.** The standard difference in the means of each covariate used in the propensity score-matched method in the SPRINT trial. **Fig. S3.** The standard difference in the means of each covariate used in the propensity score-matched method in the ACCORD trial. **Fig. S4.** Incidence rate and HRs in the intensive and standard blood pressure control groups across Achieved SBP and DBP strata for the major coronary events and all-cause death.

## Data Availability

The data that support the findings of this study are available from BioLINCC, but restrictions apply to the availability of these data, which were used under license for the current study, and so are not publicly available. Data are however available from the authors upon reasonable request and with permission from BioLINCC.

## Abbreviations

ACCORD: Action to Control Cardiovascular Risk in Diabetes
BP: Blood pressure
CI: Confidence interval
CVD: Cardiovascular disease
DBP: Diastolic blood pressure
HF: Heart failure
HR: Hazard ratios
MI: Myocardial infarction
SBP: Systolic blood pressure
SPRINT: Systolic Blood Pressure Intervention Trial
