# Supplementary material for: Clinical benefit of systolic blood pressure within the target range among patients with or without diabetes mellitus: a propensity score-matched analysis of two randomized clinical trials
Source: BMC Med. 2022 Jun 20;20:208. doi: 10.1186/s12916-022-02407-z (PMC9208196; doi:10.1186/s12916-022-02407-z)
Supplement: Supplementary file 1 — Additional file 1: Table S1. Inclusion and exclusion criteria of SPRINT and ACCORD trial. Table S2. Characteristics of patients in the intensive blood pressure control group and propensity score-matched patients from the standard blood pressure control group across the achieved diastolic blood pressure strata in the SPRINT trial. Table S3. Baseline characteristics of patients in the intensive blood pressure control group and propensity score-matched patients from the standard blood pressure control group across the achieved diastolic blood pressure strata in the ACCORD trial. Table S4. Mean standardized difference for all of the covariates used in the propensity score-matched method. Table S5. Incidence (per 100 person-years) of serious adverse events in the intensive and standard blood pressure control groups across achieved SBP and DBP strata (SPRINT trial). Fig. S1. Data exclusion in the present analysis. Fig. S2. The standard difference in the means of each covariate used in the propensity score-matched method in the SPRINT trial. Fig. S3. The standard difference in the means of each covariate used in the propensity score-matched method in the ACCORD trial. Fig. S4. Incidence rate and HRs in the intensive and standard blood pressure control groups across Achieved SBP and DBP strata for the major coronary events and all-cause death. [file 12916_2022_2407_MOESM1_ESM.docx]

**Table S1 Inclusion and exclusion criteria of SPRINT and ACCORD trial ^1, 2^**

| **ACCORD** | **SPRINT** |
| --- | --- |
| **Inclusion Criteria**   1. Age at randomization   (a) 40–79 year (inclusive) for anyone with a history of clinical CVD, or  (b) 55–79 year (inclusive) for anyone without a history of clinical CVD   1. Type 2 diabetes mellitus defined according to the 1997 ADA criteria for 3 months 2. HbA1c level 3. 7.5%–11%: (i) If on insulin <1 U/kg and on 0 or 1 oral agent or (ii) If not on insulin, and on 0, 1, or 2 oral agents, or   (b) 7.5%–9%: (i) If on insulin <1 U/kg and on 2 oral agents, (ii) If on insulin >1 U/kg and 0 oral agents, or (iii) If not on insulin and on 3 oral agents  4. Stable diabetes therapy for 3 month  5. At high risk for CVD events, defined as  (a) Presence of clinical CVD, or  (b) If no clinical CVD, evidence in the past 2 year suggesting high likelihood of CVD , or  (c) Presence of ≥2 of the following factors that increase CVD risk: LDL-C >130 mg/dL (1mg/dL=0.02586mmol/L) treated with lipid lowering medication or untreated, low HDL-C (<40 mg/dL for men and <50 mg/dL for women), SBP >140 mmHg or DBP >95 mmHg treated with BP-lowering medication or untreated, current cigarette smoking, or BMI >32  6. Inclusion criteria for BP trial:  (a) SBP 130-160 mm Hg, on 0, 1, 2, or 3 medications, or  (b) SBP 161-170 mm Hg, on 0, 1, or 2 medications, or  (c) SBP 171-180 mm Hg, on 0 or 1 medication, or  (d) SBP ≥130 mm Hg on ≥2 occasions if not currently on blood pressure lowering medication, and  (e) dipstick protein <2+, protein/Creatinine ratio <700 mg/g  **Exclusion Criteria**  1. Cardiovascular event or procedure (as defined for study entry) or hospitalization for unstable angina within past 3 months  2. A medical condition likely to limit survival to 3 years or a malignancy other than nonmelanoma skin cancer within the past 2 years  3. Weight loss >10% in past 6 months  4.Current symptomatic heart failure, history of NYHA class III or IV congestive heart failure at any time, or ejection fraction <0.25  5. History of hypoglycemic coma/seizure within past 12 month  6. Hypoglycemia requiring third-party assistance in past 3 month, with concomitant glucose 60 mg/dL (3.3 mmol/L)  7. History consistent with type 1 diabetes  8. Unwilling to do frequent capillary blood glucose self-monitoring or unwilling to inject insulin several times a day  9. BMI >45  10. Serum creatinine >1.5 mg/dL obtained within the previous 2 month  11. Transaminase >2 times the upper limit of normal or active liver disease  12. Any ongoing medical therapy with known adverse interactions with the glycemic interventions (eg, corticosteroids, protease inhibitors)  13. Any factors likely to limit adherence to interventions  14. Failure to obtain informed consent from participant  15. Currently participating in another clinical trial  16. Living in the same household as an already randomized ACCORD participant  17. Any organ transplantation  18. Pregnancy, currently trying to become pregnant, or of child-bearing potential and not practicing birth control  19. Participants with recurrent requirements for phlebotomy or transfusion of red blood cells | **Inclusion Criteria**  1. ≥50 years old  2. Systolic blood pressure  (a) SBP: 130 – 180 mm Hg on 0 or 1 medication, or  (b) SBP: 130 – 170 mm Hg on up to 2 medications, or  (c) SBP: 130 – 160 mm Hg on up to 3 medications, or  (d) SBP: 130 – 150 mm Hg on up to 4 medications  3. Risk (one or more of the following):  (a) Presence of clinical or subclinical CVD other than stroke  (b) CKD, defined as eGFR 20 – 59 ml/min/1.73m^2^ within the past 6 month  (c) Framingham Risk Score for 10-year CVD risk ≥15%  (d) Age ≥75 years.  **Exclusion Criteria**  1. Cardiovascular event or procedure (as defined for study entry) or hospitalization for unstable angina within past 3 months  2. A medical condition likely to limit survival to 3 years or a malignancy other than nonmelanoma skin cancer within the past 2 years  3. Weight loss >10% in past 6 months  4. Symptomatic heart failure within the past 6 months or left ventricular ejection fraction <0.35  5. An indication for a specific BP lowering medication  6. Known secondary cause of hypertension that causes concern regarding safety of the protocol  7. One minute standing SBP <110 mm Hg  8. Proteinuria in the following ranges (based on a measurement within the past 6 months)  (a) 24 hour urinary protein excretion ≥1 g/day, or  (b) If (a) is not available, 24 hour urinary albumin excretion ≥600  mg/day, or  (c) If (a) or (b) are not available, then spot urine protein/creatinine ratio ≥1g/g creatinine, or  (d) If (a), (b), or (c) are not available, then spot urine albumin/creatinine  ratio ≥600 mg/g creatinine, or  (e) If (a), (b), (c), or (d) are not available, then urine dipstick ≥2+ protein  9. Arm circumference too large or small to allow accurate blood pressure measurement with available devices  10. Diabetes mellitus, and  (a) Participants taking medications for diabetes in the last 12 months  (b) FPG ≥126 mg/dL, A1C ≥6.5 percent, a two-hour value in an OGTT (2-h PG) ≥200 mg/dL or a random plasma glucose concentration ≥200 mg/dL.  11. History of stroke (not CE or stenting)  12. Diagnosis of polycystic kidney disease  13. Glomerulonephritis treated with or likely to be treated with immunosuppressive therapy  14. eGFR <20 ml/min /1.73m^2^ or end-stage renal disease (ESRD)  15. Failure to obtain informed consent from participant  16. Currently participating in another clinical trial (intervention study). Note: Patient must wait until the completion of his/her activities or the completion of the other trial before being screened for SPRINT  17. Living in the same household as an already randomized SPRINT participant  18. Any organ transplant  19. Any factors judged by the clinic team to be likely to limit adherence to interventions. For example,  (a) Active alcohol or substance abuse within the last 12 month  (b) Plans to move outside the clinic catchment area in the next 2 years without the ability to transfer to another SPRINT site, or plans to be out of the study area for more than 3 months in the year following enrollment.  (c) Significant history of poor compliance with medications or attendance at clinic visits  (d) Significant concerns about participation in the study from spouse, significant other, or family members  (e) Lack of support from primary health care provider  (f) Residence too far from the study clinic site such that transportation is a barrier including persons who require transportation assistance provided by the SPRINT clinic funds for screening or randomization visits  (g) Residence in a nursing home. Persons residing in an assisted living or retirement community are eligible if they meet the other criteria.  (h) Clinical diagnosis of dementia, treatment with medications for dementia, or in the judgment of the clinician cognitively unable to follow the protocol  (i) Other medical, psychiatric, or behavioral factors that in the judgment of the Principal Investigator may interfere with study participation or the ability to follow the intervention protocol  20. Pregnancy, currently trying to become pregnant, or of child-bearing potential and not using birth control |

^1^ Clinical CVD (other than stroke) definition: a) Previous myocardial infarction, percutaneous coronary intervention, coronary artery bypass grafting, carotid endarterectomy, carotid stenting; b) Peripheral artery disease with revascularization; c) Acute coronary syndrome with or without resting ECG change, ECG changes on a graded exercise test, or positive cardiac imaging study; d) At least a 50% diameter stenosis of a coronary, carotid, or lower extremity artery; e) Abdominal aortic aneurysm ≥5 cm with or without repair.

^2^ Subclinical CVD definition: a) Coronary artery calcium score ≥400 Agatston units within the past 2 years; b) Ankle brachial index ≤0.90 within the past 2 years; c) Left ventricular hypertrophy by ECG (based on computer reading), echocardiogram report, or other cardiac imaging procedure report within the past 2 years.


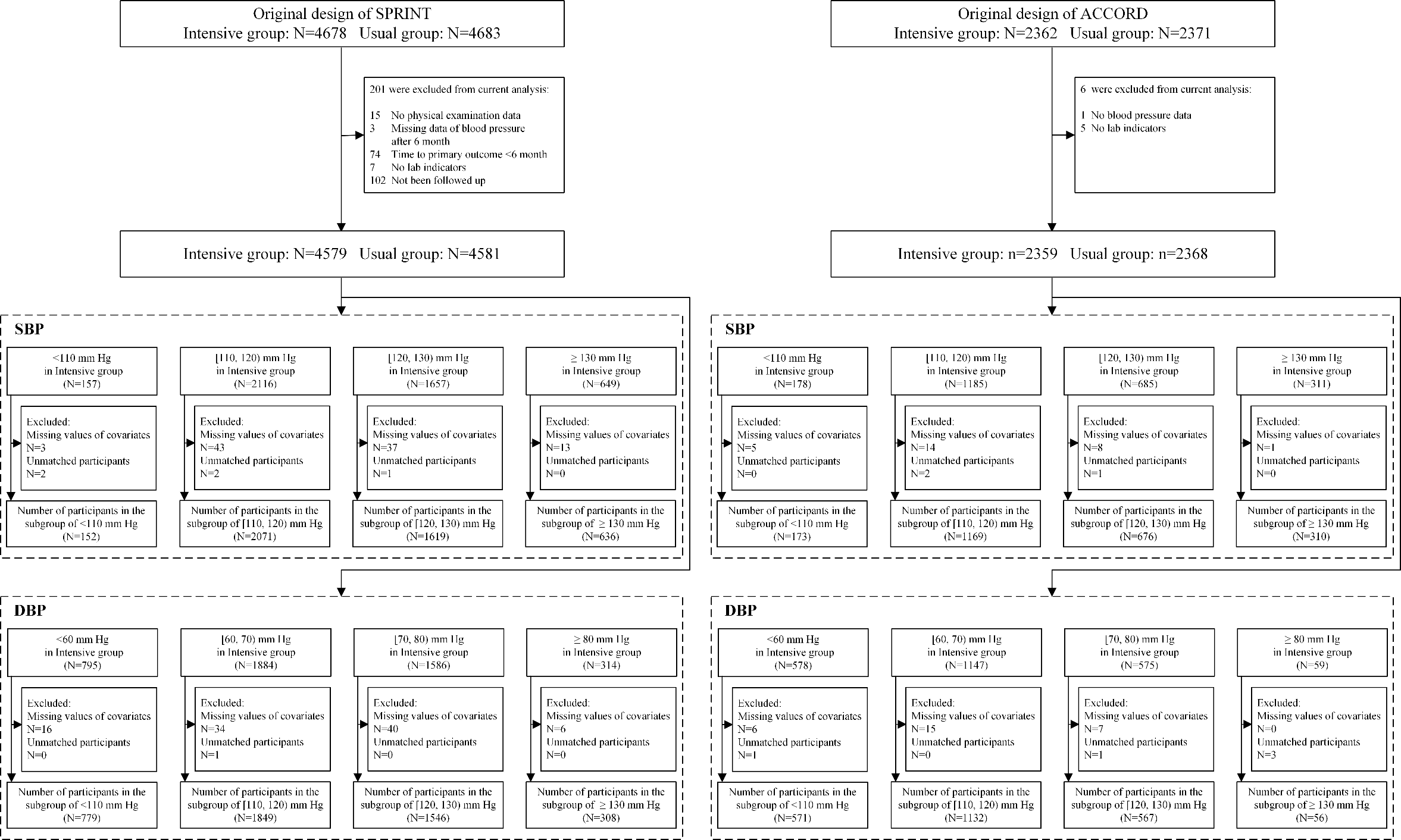
 **Figure. S1 Data exclusion in the present analysis**

| **Table S2. Characteristics of patients in the intensive blood pressure control group and propensity score-matched patients from the standard blood pressure control group across the achieved diastolic blood pressure strata in the SPRINT trial** | | | | | | | | | | | | | | |
| --- | --- | --- | --- | --- | --- | --- | --- | --- | --- | --- | --- | --- | --- | --- |
|  | Intension to treat | |  | DBP<60 mm Hg | |  | 60≤DBP<70 mm Hg | |  | 70≤DBP<80 mm Hg | |  | DBP≥80 mm Hg | |
|  | Intensive treatment | Standard treatment |  | Intensive | Usual |  | Intensive | Usual |  | Intensive | Usual |  | Intensive | Usual |
| **SPRINT** | N=4579 | N=4581 |  | N=779 | N=779 |  | N=1849 | N=1849 |  | N=1546 | N=1546 |  | N=308 | N=308 |
| **Demographics** | | | | | | | | | | | | | | |
| Age, years | 67.88±9.37 | 67.84±9.43 |  | 75.75±7.40 | 76.17±7.50 |  | 69.46±8.44 | 69.53±9.19 |  | 63.66±8.21 | 63.65±8.00 |  | 60.19±6.93 | 59.98±6.75 |
| Female | 1643(35.88) | 1603(34.99) |  | 275(35.30) | 296(38.00) |  | 663(35.86) | 646(34.94) |  | 576(37.26) | 539(34.86) |  | 103(33.44) | 107(34.74) |
| Race |  |  |  |  |  |  |  |  |  |  |  |  |  |  |
| Black | 1346(29.40) | 1389(30.32) |  | 140(17.97) | 134(17.20) |  | 473(25.58) | 405(21.90) |  | 553(35.77) | 571(36.93) |  | 149(48.38) | 145(47.08) |
| White | 2645(57.76) | 2649(57.83) |  | 555(71.25) | 573(73.56) |  | 1139(61.60) | 1198(64.79) |  | 775(50.13) | 772(49.94) |  | 117(37.99) | 114(37.01) |
| Hispanic | 493(10.77) | 473(10.32) |  | 67(8.60) | 62(7.96) |  | 201(10.87) | 204(11.03) |  | 188(12.16) | 183(11.84) |  | 33(10.71) | 43(13.96) |
| Others | 95(2.07) | 70(1.53) |  | 17(2.18) | 10(1.28) |  | 36(1.95) | 42(2.27) |  | 30(1.94) | 20(1.29) |  | 9(2.92) | 6(1.95) |
| **Medical history** |  |  |  |  |  |  |  |  |  |  |  |  |  |  |
| Clinical CVD | 755(16.49) | 758(16.55) |  | 196(25.16) | 181(23.23) |  | 326(17.63) | 364(19.69) |  | 187(12.10) | 160(10.35) |  | 31(10.06) | 39(12.66) |
| CKD | 1293(28.24) | 1287(28.09) |  | 322(41.34) | 311(39.92) |  | 524(28.34) | 568(30.72) |  | 361(23.35) | 336(21.73) |  | 67(21.75) | 59(19.16) |
| Dyslipidemia | 1941(42.39) | 2034(44.40) |  | 406(52.12) | 423(54.30) |  | 851(46.02) | 911(49.27) |  | 558(36.09) | 552(35.71) |  | 87(28.25) | 87(28.25) |
| Hypertension treatment | 4160(90.85) | 4143(90.44) |  | 729(93.58) | 727(93.32) |  | 1691(91.45) | 1675(90.59) |  | 1385(89.59) | 1377(89.07) |  | 265(86.04) | 271(87.99) |
| Aspirin treatment | 2361(51.65) | 2304(50.69) |  | 493(63.29) | 500(64.18) |  | 1012(54.73) | 1047(56.63) |  | 692(44.76) | 707(45.73) |  | 124(40.26) | 114(37.01) |
| Current smoking | 629(13.74) | 590(12.90) |  | 51(6.55) | 41(5.26) |  | 212(11.47) | 182(9,84) |  | 265(17.14) | 254(16.43) |  | 81(26.30) | 89(28.90) |
| Current drinking | 1632(35.77) | 1607(35.20) |  | 299(38.38) | 291(37.36) |  | 665(35.97) | 642(34.72) |  | 537(34.73) | 501(32.41) |  | 102(33.12) | 102(33.12) |
| **Biometric and Laboratory data** | | | | | | | | | | | | | | |
| 10-year risk for CVD, % | 24.78±12.59 | 24.78±12.44 |  | 29.62±13.46 | 29.45±13.65 |  | 25.31±12.62 | 25.40±12.90 |  | 21.92±11.25 | 22.21±11.17 |  | 21.71±10.63 | 21.61±9.55 |
| BMI, kg/m^2^ | 29.87±5.68 | 29.74±5.59 |  | 28.01±5.19 | 28.14±4.98 |  | 29.78±5.51 | 29.52±5.52 |  | 30.57±5.83 | 30.76±5.76 |  | 31.42±5.92 | 30.92±5.63 |
| SBP, mm Hg | 139.60±15.74 | 139.63±15.38 |  | 140.69±14.80 | 141.11±17.01 |  | 139.13±15.37 | 139.11±15.68 |  | 139.08±16.29 | 139.19±14.61 |  | 142.52±10.98 | 142.36±15.02 |
| DBP, mm Hg | 78.24±11.89 | 78.07±11.93 |  | 66.13±9.08 | 66.39±8.55 |  | 76.32±9.38 | 75.92±11.45 |  | 84.00±9.73 | 84.18±9.93 |  | 90.99±17.10 | 90.86±10.97 |
| BLU, mmol/L | 6.67±2.40 | 6.72±2.39 |  | 7.51±2.75 | 7.57±2.79 |  | 6.70±2.31 | 6.77±2.38 |  | 6.32±2.25 | 6.29±2.14 |  | 6.09±2.05 | 6.03±1.96 |
| Chloride, mmol/L | 102.92±2.90 | 102.93±2.84 |  | 102.89±3.28 | 102.84±3.19 |  | 102.93±2.88 | 102.94±2.76 |  | 103.00±2.73 | 102.92±2.81 |  | 102.65±2.80 | 102.83±2.60 |
| Creatinine, μmmol/L | 94.74±30.38 | 95.17±29.69 |  | 99.82±33.22 | 98.34±29.14 |  | 93.27±27.95 | 94.01±27.49 |  | 93.56±30.55 | 93.04±30.20 |  | 96.14±32.74 | 93.95±29.19 |
| Heart rate | 66.18±11.49 | 66.26±11.59 |  | 61.75±10.28 | 61.69±10.23 |  | 65.40±10.82 | 65.21±11.20 |  | 68.07±11.59 | 67.98±11.34 |  | 71.49±12.56 | 70.42±12.65 |
| eGFR, ml/min/1.73m^2^ | 71.90±20.68 | 71.69±20.44 |  | 65.30±19.55 | 65.51±20.68 |  | 71.86±20.29 | 70.78±20.11 |  | 74.28±20.62 | 75.28±20.22 |  | 75.91±21.17 | 76.64±19.63 |
| Glucose, mmol/L | 5.54±0.77 | 5.53±0.75 |  | 5.55±0.68 | 5.59±0.70 |  | 5.56±0.77 | 5.58±0.84 |  | 5.51±0.80 | 5.51±0.74 |  | 5.48±0.84 | 5.44±0.77 |
| HDL-C, mmol/L | 1.37±0.37 | 1.37±0.38 |  | 1.39±0.38 | 1.40±0.36 |  | 1.39±0.36 | 1.36±0.36 |  | 1.36±0.38 | 1.36±0.37 |  | 1.34±0.33 | 1.36±0.35 |
| LDL-C, mmol/L | 2.91±0.92 | 2.90±0.90 |  | 2.68±0.84 | 2.69±0.82 |  | 2.86±0.88 | 2.83±0.85 |  | 3.05±0.95 | 3.06±0.90 |  | 3.14±0.94 | 3.24±0.92 |
| Potassium, mmol/L | 4.21±0.44 | 4.20±0.45 |  | 4.28±0.49 | 4.28±0.45 |  | 4.20±0.41 | 4.21±0.44 |  | 4.17±0.43 | 4.18±0.48 |  | 4.17±0.41 | 4.20±0.56 |
| Sodium, mmol/L | 140.12±2.46 | 140.15±2.42 |  | 140.09±2.77 | 139.97±2.92 |  | 140.20±2.47 | 140.18±2.37 |  | 140.12±2.24 | 140.09±2.38 |  | 139.90±2.54 | 140.10±2.21 |
| TC, mmol/L | 4.92±1.07 | 4.91±1.06 |  | 4.65±1.01 | 4.66±0.96 |  | 4.86±1.02 | 4.82±0.98 |  | 5.05±1.09 | 5.05±1.04 |  | 5.14±1.07 | 5.26±1.04 |
| Triglycerides, mmol/L | 1.37±0.95 | 1.40±1.04 |  | 1.23±0.62 | 1.21±0.56 |  | 1.31±0.66 | 1.33±0.66 |  | 1.38±0.68 | 1.35±0.67 |  | 1.40±0.73 | 1.40±0.76 |
| **Post-baseline characteristics** | | | | | | | | | | | | | | |
| Achieved SBP, mm Hg | 121.72±8.78 | 135.51±7.45 |  | 120.91±8.19 | 136.56±8.00 |  | 120.04±7.41 | 135.55±7.58 |  | 122.16±8.26 | 135.76±7.61 |  | 131.63±12.84 | 134.36±7.62 |
| Achieved DBP, mm Hg | 67.98±8.43 | 75.11±9.17 |  | 55.58±3.75 | 66.20±7.77 |  | 65.35±2.84 | 73.62±8.91 |  | 74.07±2.79 | 79.99±7.44 |  | 84.44±5.07 | 82.64±6.63 |

BLU, Blood urea nitrogen; BMI, Body mass index; CKD, Chronic Kidney Disease; CVD, Cardiovascular disease; DBP, Diastolic blood pressure; eGFR, Estimated glomerular filtration rate; HLD-C, High-density lipoprotein cholesterol; LDL-C, Low-density lipoprotein cholesterol; SBP, Systolic blood pressure; TC, Total cholesterol.

Categorical variables are reported as percentage with the characteristic. Continuous variables are reported as mean±SD.

Baseline covariables list in the table are added into the logistic model to calculate propensity score of each patient from the SPRINT trial.

| **Table S3. Baseline characteristics of patients in the intensive blood pressure control group and propensity score-matched patients from the standard blood pressure control group across the achieved diastolic blood pressure strata in the ACCORD trial** | | | | | | | | | | | | | | |
| --- | --- | --- | --- | --- | --- | --- | --- | --- | --- | --- | --- | --- | --- | --- |
|  | Intension to treat | |  | DBP<60 mm Hg | |  | 60≤DBP<70 mm Hg | |  | 70≤DBP<80 mm Hg | |  | DBP≥80 mm Hg | |
|  | Intensive treatment | Standard treatment |  | Intensive | Usual |  | Intensive | Usual |  | Intensive | Usual |  | Intensive | Usual |
| **ACCORD** | N=2359 | N=2368 |  | N=571 | N=571 |  | N=1132 | N=1132 |  | N=567 | N=567 |  | N=56 | N=56 |
| **Demographics** | | | | | | | | | | | | | | |
| Age, years | 62.72±6.60 | 62.75±6.76 |  | 66.04±6.39 | 66.70±6.65 |  | 62.61±6.12 | 62.55±6.68 |  | 59.85±6.53 | 59.87±5.31 |  | 60.57±6.56 | 59.96±6.60 |
| Female | 1127(47.77) | 1129(47.68) |  | 241(42.21) | 233(40.81) |  | 541(47.79) | 537(47.44) |  | 293(51.68) | 296(52.20) |  | 32(57.14) | 26(46.43) |
| Race |  |  |  |  |  |  |  |  |  |  |  |  |  |  |
| Black | 546(23.15) | 577(24.37) |  | 101(17.69) | 110(19.26) |  | 239(21.11) | 249(22.00) |  | 173(30.51) | 169(29.81) |  | 23(41.07) | 15(26.79) |
| White | 1411(59.81) | 1368(57.77) |  | 393(68.83) | 368(64.45) |  | 704(62.19) | 703(62.19) |  | 270(47.62) | 263(46.38) |  | 24(42.86) | 30(53.57) |
| Hispanic | 160(6.78) | 170(7.18) |  | 31(5.43) | 39(6.83) |  | 64(5.65) | 68(6.01) |  | 57(10.05) | 57(10.05) |  | 8(14.29) | 7(12.50) |
| Others | 242(10.26) | 253(10.68) |  | 54(9.46) | 46(8.06) |  | 125(11.04) | 112(9.89) |  | 67(11.82) | 78(13.76) |  | 3(5.36) | 2(3.57) |
| Randomized glycemic treatment | 1177(49.89) | 1193(50.38) |  | 289(50.61) | 315(55.17) |  | 570(50.35) | 544(48.06) |  | 278(49.03) | 299(52.73) |  | 24(42.86) | 27(48.21) |
| **Medical history** | | | | | | | | | | | | | | |
| Clinical CVD | 803(34.04) | 788(33.28) |  | 246(43.08) | 226(39.58) |  | 341(30.12) | 346(30.57) |  | 160(28.22) | 181(31.92) |  | 18(32.14) | 22(39.29) |
| Clinical heart disease | 658(27.89) | 657(27.74) |  | 210(36.78) | 191(33.45) |  | 270(23.85) | 274(24.20) |  | 152(26.81) | 129(22.75) |  | 14(25.00) | 17(30.36) |
| Dyslipidemia | 1629(69.06) | 1670(70.52) |  | 395(69.18) | 365(63.92) |  | 793(70.05) | 830(73.32) |  | 380(67.02) | 396(69.84) |  | 39(69.64) | 36(64.29) |
| Hypertension treatment | 2082(88.26) | 2068(87.33) |  | 509(89.14) | 509(89.14) |  | 989(87.37) | 995(87.90) |  | 501(88.36) | 492(86.77) |  | 51(91.07) | 45(80.36) |
| Dyslipidemia treatment | 1906(80.80) | 1892(79.90) |  | 484(84.76) | 473(82.84) |  | 929(82.07) | 931(82.24) |  | 426(75.13) | 437(77.07) |  | 42(75.00) | 48(85.71) |
| Current smoking | 971(41.16) | 977(41.26) |  | 287(50.26) | 275(48.16) |  | 467(41.25) | 466(41.17) |  | 189(33.33) | 199(35.10) |  | 16(28.57) | 13(23.21) |
| Current drinking | 561(23.78) | 544(22.97) |  | 132(23.12) | 123(21.54) |  | 278(24.56) | 257(22.70) |  | 132(23.28) | 135(23.81) |  | 11(19.64) | 17(30.36) |
| **Biometric and Laboratory data** | | | | | | | | | | | | | | |
| BMI, kg/m^2^ | 32.19±5.60 | 32.10±5.38 |  | 31.13±5.25 | 30.85±4.95 |  | 32.35±5.69 | 32.60±5.24 |  | 32.71±5.52 | 32..41±5.25 |  | 33.24±6.53 | 33.33±4.53 |
| SBP, mm Hg | 139.02±16.12 | 139.34±15.54 |  | 138.67±16.65 | 139.32±16.46 |  | 139.08±16.31 | 138.14±15.23 |  | 138.49±14.96 | 138.26±13.91 |  | 144.52±14.66 | 144.84±13.26 |
| DBP, mm Hg | 75.93±10.56 | 75.98±10.23 |  | 67.71±8.78 | 67.52±8.41 |  | 76.41±8.94 | 76.58±10.28 |  | 81.91±9.22 | 82.02±8.89 |  | 88.43±9.24 | 89.73±10.79 |
| Waist, cm | 106.02±14.08 | 105.36±13.26 |  | 105.03±14.00 | 104.51±13.23 |  | 106.44±14.35 | 107.03±13.50 |  | 106.08±13.31 | 105.82±12.06 |  | 106.89±15.67 | 107.48±11.78 |
| Creatinine, μmmol/L | 79.22±21.05 | 79.22±20.85 |  | 81.71±20.47 | 83.77±20.48 |  | 78.66±21.03 | 79.29±21.50 |  | 78.48±21.81 | 75.63±20.00 |  | 74.98±16.77 | 75.61±16.07 |
| CPK, mg/dL | 137.45±119.35 | 146.51±145.12 |  | 136.69±121.84 | 142.59±113.22 |  | 138.14±119.44 | 136.69±115.71 |  | 138.85±120.67 | 138.67±109.38 |  | 130.79±103.60 | 112.80±66.05 |
| Egfr, ml/min/1.73m^2^ | 91.57±30.30 | 91.63±27.13 |  | 87.70±24.83 | 85.64±24.75 |  | 92.23±34.90 | 91.95±29.03 |  | 93.44±25.77 | 96.85±25.67 |  | 95.99±18.20 | 93.78±19.15 |
| Glucose, mmol/L | 9.86±3.23 | 9.70±3.23 |  | 9.50±2.99 | 9.56±3.37 |  | 9.96±3.24 | 9.98±3.32 |  | 9.99±3.46 | 10.04±3.36 |  | 9.82±2.86 | 9.75±2.78 |
| HDL-C, mmol/L | 1.19±0.34 | 1.20±0.36 |  | 1.20±0.33 | 1.23±0.38 |  | 1.18±0.35 | 1.17±0.35 |  | 1.20±0.34 | 1.20±0.35 |  | 1.20±0.37 | 1.20±0.51 |
| LDL-C, mmol/L | 2.87±0.97 | 2.81±0.93 |  | 2.83±0.93 | 2.71±0.91 |  | 2.83±0.97 | 2.79±0.93 |  | 2.98±0.99 | 3.02±1.03 |  | 2.97±0.82 | 2.86±1.01 |
| Potassium, mmol/L | 4.47±0.47 | 4.47±0.57 |  | 4.56±0.54 | 4.53±0.47 |  | 4.46±0.43 | 4.45±0.43 |  | 4.43±0.43 | 4.41±0.42 |  | 4.35±0.50 | 4.37±0.42 |
| TC, mmol/L | 5.02±1.17 | 4.95±1.15 |  | 4.90±1.08 | 4.78±1.13 |  | 5.03±1.21 | 4.95±1.12 |  | 5.11±1.15 | 5.15±1.20 |  | 5.15±0.92 | 5.19±1.38 |
| Triglycerides, mmol/L | 2.14±1.96 | 2.10±2.03 |  | 1.92±1.50 | 1.86±1.56 |  | 2.29±2.24 | 2.22±1.88 |  | 2.09±1.76 | 2.15±1.89 |  | 2.31±1.90 | 2.73±2.74 |
| **Post-baseline characteristics** |  |  |  |  |  |  |  |  |  |  |  |  |  |  |
| Achieved SBP, mm Hg | 120.43±9.66 | 133.83±9.40 |  | 118.84±8.12 | 133.08±9.13 |  | 119.35±8.60 | 133.60±9.64 |  | 122.48±10.05 | 134.49±9.08 |  | 138.18±16.80 | 134.54±10.21 |
| Achieved DBP, mm Hg | 65.39±7.48 | 71.31±8.01 |  | 56.08±3.31 | 64.98±7.07 |  | 64.97±2.89 | 72.34±8.13 |  | 73.66±2.63 | 75.87±7.03 |  | 84.20±4.61 | 78.05±8.64 |

BMI, Body mass index; CPK, Creatine phosphokinase; CVD, Cardiovascular disease; DBP, Diastolic blood pressure; eGFR, Estimated glomerular filtration rate; HLD-C, High-density lipoprotein cholesterol; LDL-C, Low-density lipoprotein cholesterol; SBP, Systolic blood pressure; TC, Total cholesterol.

Categorical variables are reported as percentage with the characteristic. Continuous variables are reported as mean±SD.

Baseline covariables list in the table are added into the logistic model to calculate propensity score of each patient from the ACCORD trial.

| **Table S4. Mean standardized difference for all of the covariates used in the propensity score-matched method** | | | | | |
| --- | --- | --- | --- | --- | --- |
|  | SPRINT | |  | ACCORD | |
|  | Matched | Unmatched |  | Matched | Unmatched |
| **SBP Strata** |  |  |  |  |  |
| SBP<110 mm Hg | 6.8 | 13.7 |  | 5.2 | 13.5 |
| 110≤SBP<120 mm Hg | 2.0 | 5.5 |  | 2.7 | 5.1 |
| 120≤SBP<130 mm Hg | 1.9 | 3.5 |  | 2.9 | 4.8 |
| SBP≥130 mm Hg | 2.8 | 12.8 |  | 4.2 | 15.0 |
| **DBP Strata** |  |  |  |  |  |
| DBP<60 mm Hg | 4.2 | 24.3 |  | 4.8 | 16.0 |
| 60≤DBP<70 mm Hg | 2.2 | 4.6 |  | 2.3 | 4.0 |
| 70≤DBP<80 mm Hg | 2.1 | 12.7 |  | 2.0 | 13.0 |
| DBP≥80 mm Hg | 5.3 | 25.6 |  | 9.7 | 24.3 |

Propensity score was calculated by fitting logistic regression model with adding the variables of age, sex, race, history of clinical CVD, history of CKD, history of dyslipidemia, history of hypertensive treatment, history of aspirin treatment, current smoking, current drinking, 10-year risk for CVD, BMI, SBP, DBP, BLU, chloride, serum Creatine, heart rate, eGFR, glucose, HDL-C, LDL-C, potassium, sodium, total cholesterol, triglycerides in SPRINT study; Age, sex, race, history of clinical CVD, history of heart disease, history of dyslipidemia, history of hypertensive treatment, history of dyslipidemia treatment, current smoking, current drinking, BMI, SBP, DBP, waist, serum Creatine, CPK, eGFR, glucose, HDL-C, LDL-C, potassium, total cholesterol, triglycerides in the ACCORD trial.

BLU, Blood urea nitrogen; BMI, Body mass index; CKD, Chronic Kidney Disease; CVD, Cardiovascular disease; DBP, Diastolic blood pressure; eGFR, Estimated glomerular filtration rate; HLD-C, High-density lipoprotein cholesterol; LDL-C, Low-density lipoprotein cholesterol; SBP, Systolic blood pressure; CPK: Creatine phosphokinase.

| **Table S5. Incidence (per 100 person-years) of serious adverse events in the intensive and standard blood pressure control groups across achieved SBP and DBP strata (SPRINT trial)** | | | | | | | | | | | |
| --- | --- | --- | --- | --- | --- | --- | --- | --- | --- | --- | --- |
|  | SBP<110 mm Hg | |  | 110≤SBP<120 mm Hg | |  | 120≤SBP<130 mm Hg | |  | SBP≥130 mm Hg | |
|  | Intensive | Standard |  | Intensive | Standard |  | Intensive | Standard |  | Intensive | Standard |
| Any serious adverse event | 14.73(11.30, 19.40) | 13.26(9.89, 18.03) |  | 12.33(11.46, 13.28) | 13.24(12.01, 14.63) |  | 15.95(14.77, 17.22) | 14.26(12.91, 15.77) |  | 21.63(19.30, 24.28) | 18.76(16.47, 21.41) |
| **Serious adverse events associated with:** | |  |  |  |  |  |  |  |  |  |  |
| Hypotension | 0.43(0.09, 4.29) | 0.86(0.32, 3.08) |  | 0.54(0.39, 0.76) | 0.24(0.14, 0.45) |  | 0.92(0.70, 1.25) | 0.26(0.15, 0.47) |  | 1.18(0.78, 1.88) | 0.81(0.45, 1.60) |
| Syncope | 0.21(-, -) | 1.08(0.37, 4.40) |  | 0.57(0.42, 0.79) | 0.43(0.28, 0.69) |  | 0.77(0.56, 1.07) | 0.50(0.32, 0.82) |  | 1.34(0.91, 2.06) | 0.81(0.45, 1.61) |
| Electrolyte abnormality | 0.87(0.33, 3.08) | 0.64(0.20, 3.13) |  | 0.55(0.40, 0.78) | 0.74(0.50, 1.14) |  | 1.24(0.98, 1.61) | 0.90(0.63, 1.31) |  | 1.98(1.44, 2.81) | 1.59(1.07, 2.48) |
| Acute kidney injury or | 1.08(0.46, 3.19) | 1.51(0.73, 3.62) |  | 0.73(0.56, 0.98) | 0.72(0.51, 1.07) |  | 1.44(1.15, 1.83) | 0.88(0.63, 1.26) |  | 3.24(2.51, 4.26) | 0.86(0.52, 1.56) |
| acute kidney failure |  |  |  |  |  |  |  |  |  |  |  |
|  | DBP<60 mm Hg | |  | 60<DBP<70 mm Hg | |  | 70<DBP<80 mm Hg | |  | DBP≥80 mm Hg | |
|  | Intensive | Usual |  | Intensive | Usual |  | Intensive | Usual |  | Intensive | Usual |
| Any serious adverse event | 18.30(16.51, 20.32) | 17.37(15.17, 19.93) |  | 14.52(13.50, 15.63) | 14.76(13.40, 16.28) |  | 13.60(12.50, 14.80) | 12.27(10.95, 13.77) |  | 14.28(11.75, 17.46) | 12.13(9.62, 15.42) |
| **Serious adverse events associated with:** | |  |  |  |  |  |  |  |  |  |  |
| Hypotension | 0.65(0.41, 1.12) | 0.41(0.18, 1.16) |  | 0.75(0.56, 1.02) | 0.40(0.26, 0.64) |  | 0.78(0.58, 1.09) | 0.34(0.18, 0.73) |  | 0.92(0.47, 2.07) | 0.41(0.11, 2.59) |
| Syncope | 0.74(0.47, 1.21) | 0.49(0.22, 1.37) |  | 0.76(0.57, 1.04) | 0.55(0.35, 0.93) |  | 0.72(0.52, 1.02) | 0.34(0.21, 0.62) |  | 0.46(0.17, 1.63) | 0.83(0.36, 2.40) |
| Electrolyte abnormality | 1.83(1.37, 2.50) | 0.99(0.59, 1.78) |  | 0.76(0.56, 1.03) | 0.87(0.57, 1.38) |  | 0.78(0.57, 1.10) | 0.55(0.34, 0.92) |  | 1.39(0.81, 2.62) | 0.52(0.22, 1.55) |
| Acute kidney injury or | 1.66(1.23, 2.29) | 1.03(0.65, 1.73) |  | 1.33(1.07, 1.68) | 0.74(0.50, 1.14) |  | 1.14(0.88, 1.50) | 0.53(0.33, 0.89) |  | 1.16(0.63, 2.34) | 0.62(0.28, 1.64) |
| acute kidney failure |  |  |  |  |  |  |  |  |  |  |  |

Incidence rates of the indicated adverse events expressed as number of events per 100 person-years of follow-up.


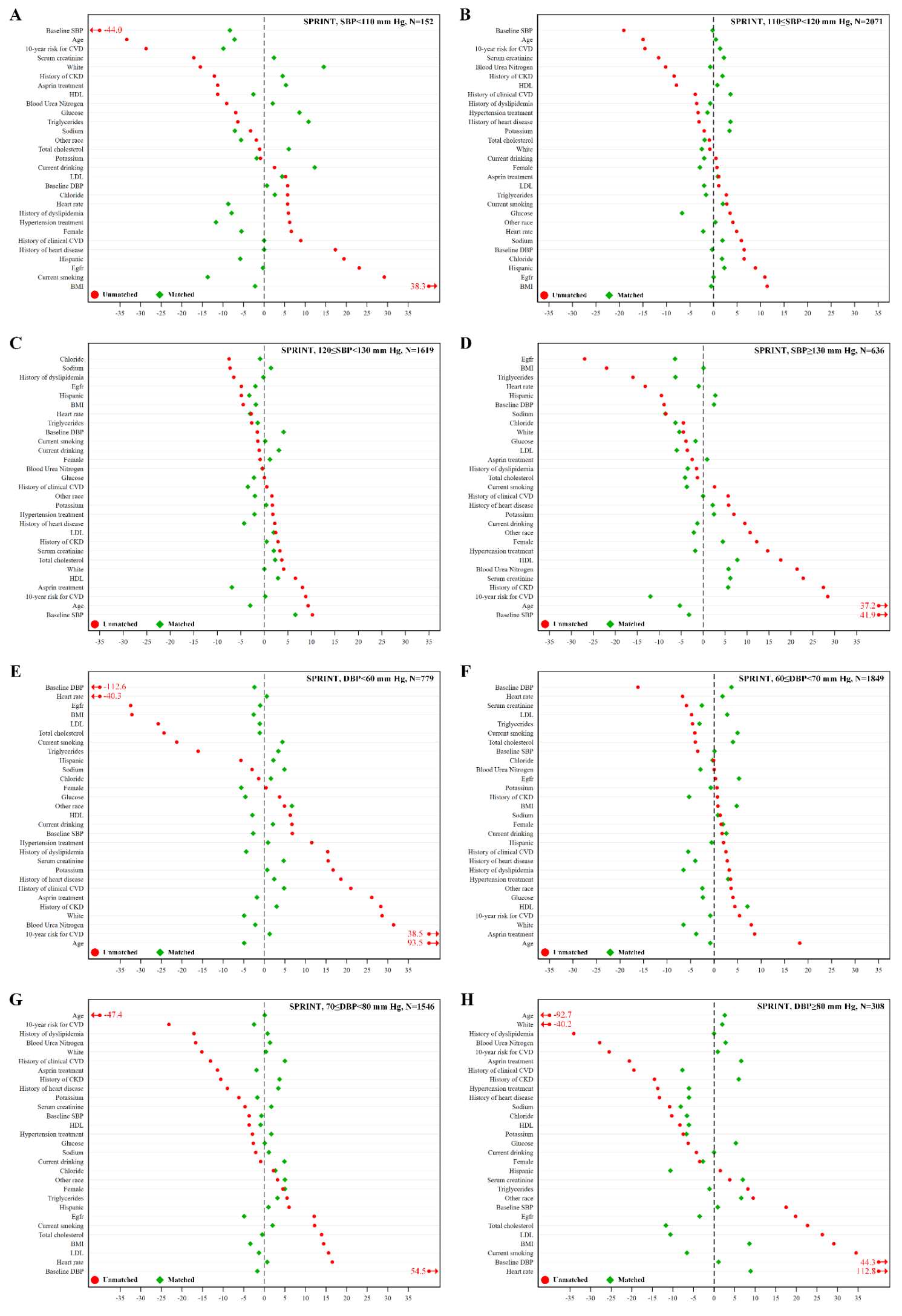


**Figure S2 The standard difference in the means of each covariate used in the propensity score-matched method in the SPRINT trial**


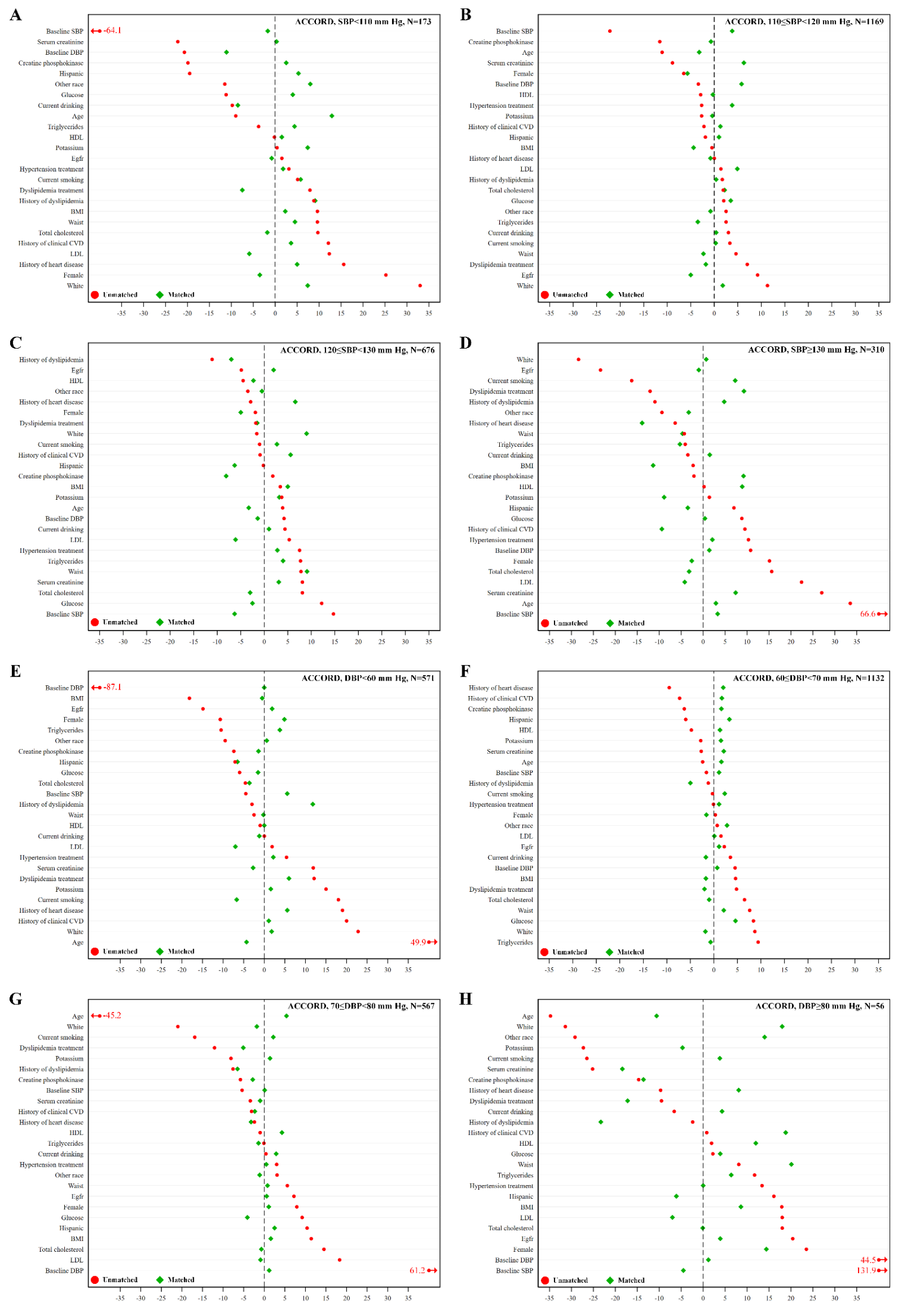


**Figure S3 The standard difference in the means of each covariate used in the propensity score-matched method in the ACCORD trial**


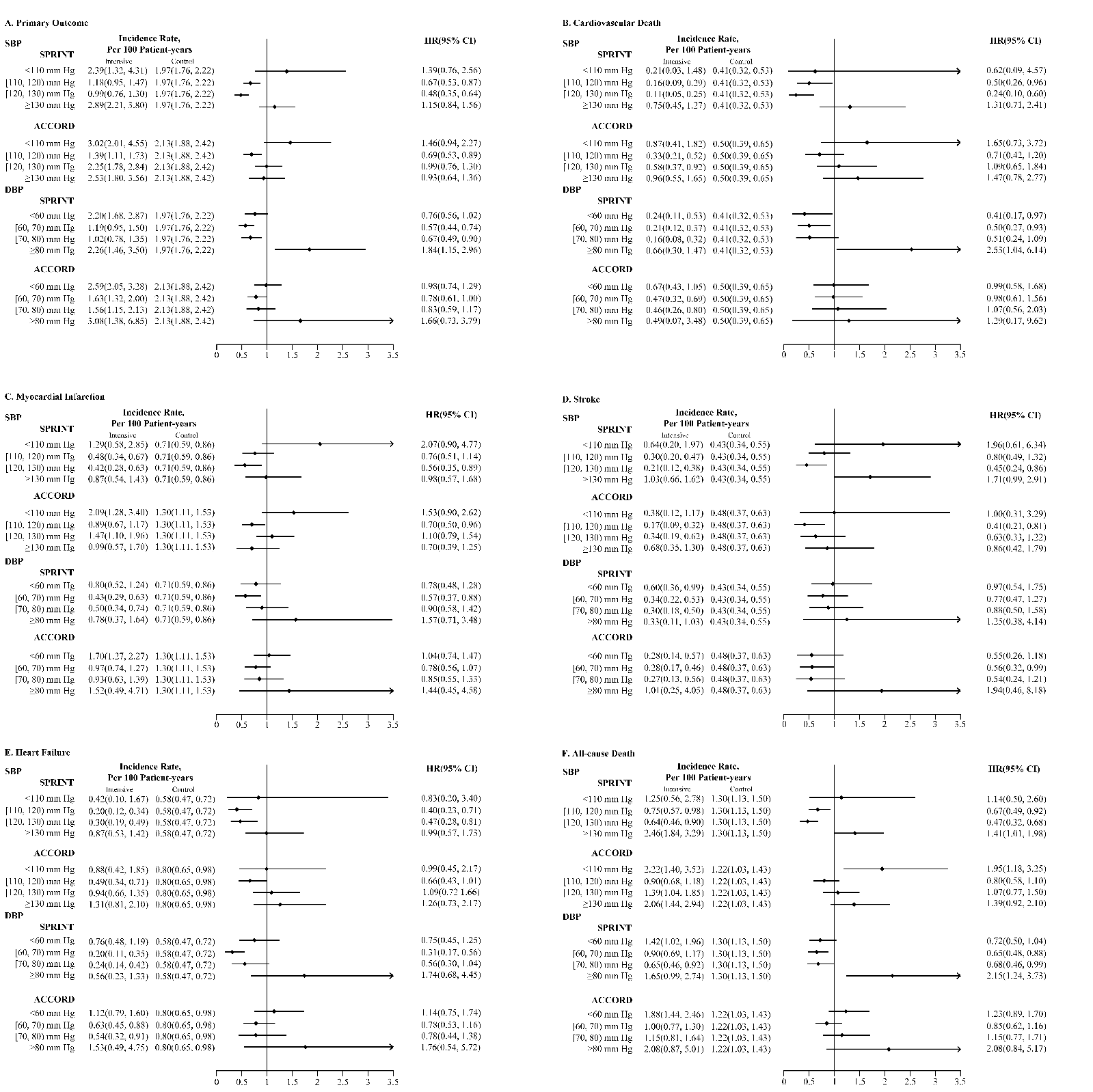


**Figure S4 Incidence rate and HRs in the intensive and standard blood pressure control groups across Achieved SBP and DBP strata for the major coronary events and all-cause death**

Incidence rate per 100 patient-years and adjusted HRs of intensive blood pressure treatment effect, compared with all patients in the standard blood pressure control, in SPRINT and ACCORD studies according to levels of achieved SBP and DBP with (A) primary outcome, (B) cardiovascular death, (C) myocardial infarction, (D) stroke, (E) heart failure, (F) all-cause death.

HRs calculations were adjusted for the variables of age, sex, race, history of clinical CVD, history of hypertensive treatment, history of dyslipidemia treatment, current smoking, current drinking, BMI, SBP, DBP, eGFR, glucose, HDL-C, LDL-C, potassium, total cholesterol, triglycerides.

BMI, Body mass index; CVD, Cardiovascular disease; DBP, Diastolic blood pressure; eGFR, Estimated glomerular filtration rate; HLD-C, High-density lipoprotein cholesterol; LDL-C, Low-density lipoprotein cholesterol; SBP, Systolic blood pressure.
